# Collargoi Enemata in Septic Affections

**Published:** 1908-10

**Authors:** 


### Collargol Enemata in Septic Affections.

Curt Seidel (*Deutsche med. Wochenschrift,* July 30, 1908,) says: the introduction of collargol into the system by the inunction of unguentum Credé develops a gradual effect and its employment is limited in cases of emaciation and in painful affections. Hence this method is indicated in mild to medium severe or localised and in chronic or subchronic infections. The intravenous injection of collargol, though the sovereign method in grave cases where a rapid and intensive effect is necessary, is often technically difficult. In such cases collargol is often advantageously administered per rectum as originally proposed by Loebl (Schlesinger's division of the Vienna Franz-Joseph Spital) in puerperal sepsis and endorsed by Witthauer in joint rheumatism.

Given by enema, collargol is, of course, less rapidly absorbed into the blood and tissue fluids than when injected intravenously; moreover, the entire quantity is rarely absorbed. Hence a correspondingly larger dose must be used per rectum.

Collargolum enemata have been given by Seidel in over 100 extremely severe cases, such as were formerly treated with collargol intravenously. He gives the case histories of eight typical ones. The treatment almost never fails, not even in very grave cases, if it is only pushed with the necessary vigor and persistence—a fact which Seidel desires to impress on those physicians who have spoken disparagingly of collargol after they saw no result from their timid and small doses. Collargol is, of course, no panacea; just as a rheumatism or a malaria can not be cured with a single dose of a salicylate or quinine, just so is it irrational to expect a sudden recovery from a sepsis or pyemia after one insufficient or delayed administration of collargol.

Seidel's cases showed that collargol enemata have a material, if not a decisive, influence on the favorable course of severe affections. They produced a rapid improvement in the general condition, return of sleep and appetite, and remission of fever, more or less quickly in accordance with the severity of the case. Self-deceptions are wholly excluded with one who has thoroughly studied collargol therapy.

Seidel gives the following directions for collargol enemata: (1) A cleansing clyster with warm soap suds. (2) Fifteen minutes after the rejection of the clyster and passage of the fecal residue, a careful irrigation with sodium chloride solution is made, to remove intestinal mucus. (3) Fifteen minutes thereafter an enema of 30 to 75 grains of collargol in two to four ounces of warm boiled water, once or twice daily. This is for severe cases; the dose in milder or chronic ones is 15 to 30 grains. (4) Upon the appearance of the effect, the dose is diminished, but the enemas should be continued for at least two weeks. In case of recrudescence, immediate resumption of the treatment, if the relapse is not due to abscess formation or other local process.

Rectal application of collargol, which leaves nothing to be desired in simplicity and convenience, is indicated not only in septic processes, but also in infectious diseases and mixed infections. He enumerates rheumatism, pneumonia, typhoid fever, septic scarlatina, septic diphtheria, anthrax, leprosy, cerebrospinal meningitis, dysentery, infectious gastrointestinal catarrh (particularly in children), and other general and local bacterial invasions such as angina, phlegmon, erythema nodosum, erysipelas and septic nephritis.

